# From structure to structural dynamics: Ahmed Zewail's legacy

**DOI:** 10.1063/1.4998243

**Published:** 2017-08-18

**Authors:** Majed Chergui, John Meurig Thomas

**Affiliations:** 1 Ecole Polytechnique Fédérale de Lausanne, Laboratoire de Spectroscopie Ultrarapide and Lausanne Centre for Ultrafast Science (LACUS), ISIC, Faculté des Sciences de Base, Station 6, CH-1015 Lausanne, Switzerland; 2 Department of Materials Science and Metallurgy, University of Cambridge, 27 Charles Babbage Road, Cambridge CB30FS, United Kingdom

## Abstract

In this brief tribute to Ahmed Zewail, we highlight and place in the historical context, several of the major achievements that he and his colleagues have made in Femtochemistry (of which he was the principal instigator) and his introduction of ultrafast electron scattering, diffraction, microscopy and spectroscopy. By achieving a sub-picosecond temporal resolution, coupled with a picometer spatial resolution, he revolutionised our understanding of the corpus of chemical, physical, biological and materials science systems.

## I. HISTORICAL BACKGROUND

The field of structural dynamics is the merger of atomic scale structural methods with the atomic scale temporal ones. The former preceded the latter by almost a century. The discovery of X-rays by Röntgen in 1895, shortly followed in 1897 by that of the electron by Thomson, sparked off an ever-continuing chain of scientific breakthroughs in the first three decades of the 20th century that would mark the birth of structural science. In parallel, efforts were being pursued aimed at time resolving chemical reactions, which would lead to the birth of Femtochemistry at the end of the 20th century. In this introduction, we briefly review the main developments in both types of methods.

### A. The spatial dimension

The systematic investigation of the characteristic X-rays (in emission and absorption) by Barkla led to the discovery of the famous X-ray absorption edges (interestingly, this discovery preceded X-ray diffraction).^1,2^ Shortly after, in the period between 1912 and 1913, Ewald, von Laue, Bragg and Bragg heralded the birth of a new era by establishing the laws of X-ray diffraction from crystals and their structural determination with atomic resolution.^3–7^

In the real world, order is often disrupted by defects and/or thermal motion, even at absolute zero temperature. Therefore, the representation of a crystal as a highly ordered arrangement of atoms and molecules with perfect periodicity in three dimensions is idealistic. Already in the 1910s, Debye got interested in the effects of thermal motions and disorder. He demonstrated how these led to a decrease of the diffraction intensities of Bragg reflections, especially at high scattering angles.^8^ This was the birth of Debye-Scherrer scattering or powder diffraction and of diffuse scattering, which apply to disordered media. Debye's work already implied an awareness about resolving atomic motion, but it would take several decades to make this possible, as discussed below.

In parallel to the discoveries concerning scattering phenomena, work was pursued to understand and interpret X-ray absorption and emission spectra, as the development of quantum mechanics was unfolding. Moseley^9^ derived an empirical law concerning the characteristic x-rays that are emitted by atoms, and Siegbahn^10–13^ developed new apparata and methods to rigorously analyse X-ray spectra and use them for the chemical identification of elements and analysis of electronic structure. As a structural tool, X-ray absorption spectroscopy would have to wait several decades of theoretical developments^14^ before the origin of the modulations that appear in the above-edge region of the spectrum of an atom in a molecular or crystalline edifice could be understood. These above-edge modulations, called X-ray absorption near-edge structure (XANES) and extended X-ray absorption fine structure (EXAFS), finally received a consistent interpretation through the works of Sayers, Stern and Lytle,^15,16^ making EXAFS in particular (whose treatment is easier), an important element-selective tool in Materials Science, Biology and Chemistry.

As far as the electron is concerned, a turning point was the enunciation in 1924 of the wave-particle duality by de Broglie, shortly after followed by the famous electron diffraction experiments by Davisson and Germer and by Thomson (J. J. Thomson's son) in 1927 (for this discovery, Thomson and Davisson received the Nobel Prize for Physics in 1937).

In 1928, Hans Bethe, then a student of Sommerfeld, discussed the process of multiple scattering inherent to the diffraction experiments. X-ray photons are scattered by the electron distribution (Thomson scattering), while electrons are scattered by both the atomic nuclei and the electron distribution. Because of Coulomb scattering, electron-scattering cross-sections are some five to six orders of magnitude larger than those of X-rays. It was this feature that prompted Mark and Wierl in 1930^17^ to use electrons (instead of X-rays) to elucidate gas-phase molecular structures. They produced a diffraction pattern from the vapour of CCl_4_ that was more distinct than similar X-ray scattering exposures obtained earlier by Debye and coworkers,^18,19^ and required a fraction of the exposure time (1 s compared to 20 h for the X-ray pattern).

The first electromagnetic lens was developed in 1926 by Busch.^20^ Soon after in 1931, the physicist Ernst Ruska and the electrical engineer Knoll constructed the prototype electron microscope, capable of four-hundred-power magnification; this represented the first demonstration of the principles of electron microscopy.^21^ Two years later, Ruska built an electron microscope that exceeded the resolution attainable with an optical (light) microscope.^22^ The interest in microscopy to investigate biological specimens was already clear.^23^ In 1937, von Ardenne pioneered the scanning electron microscope.^24^ Although contemporary transmission electron microscopes are capable of two million-power magnification, as scientific instruments, they remain based on Ruska's prototype.

In 1948, Gabor introduced electron holography, and in 1951,^25^ the first imaging of individual atoms by field emission electron microscopy was achieved by Müller.^26^ All these discoveries established X-ray and electron diffraction and microscopy as the tools of choice to probe matter at the atomic scale resolution of space, i.e., the Å. It is fair to say that while several spectacular improvements would come in the following decades, the stage was set in the 1910–1930 period for the static structural determination of assemblies of atoms (crystals, molecules, proteins). Ahmed Zewail frequently used to quote Francis Crick's well-known dictum: “*If you want to understand function, study structure.*” But, he would quickly emphasize: “*If you want to study function, it is necessary to study the time-dependent structure, i.e., the dynamics.*”

### B. The time dimension

**FIG. 1.**
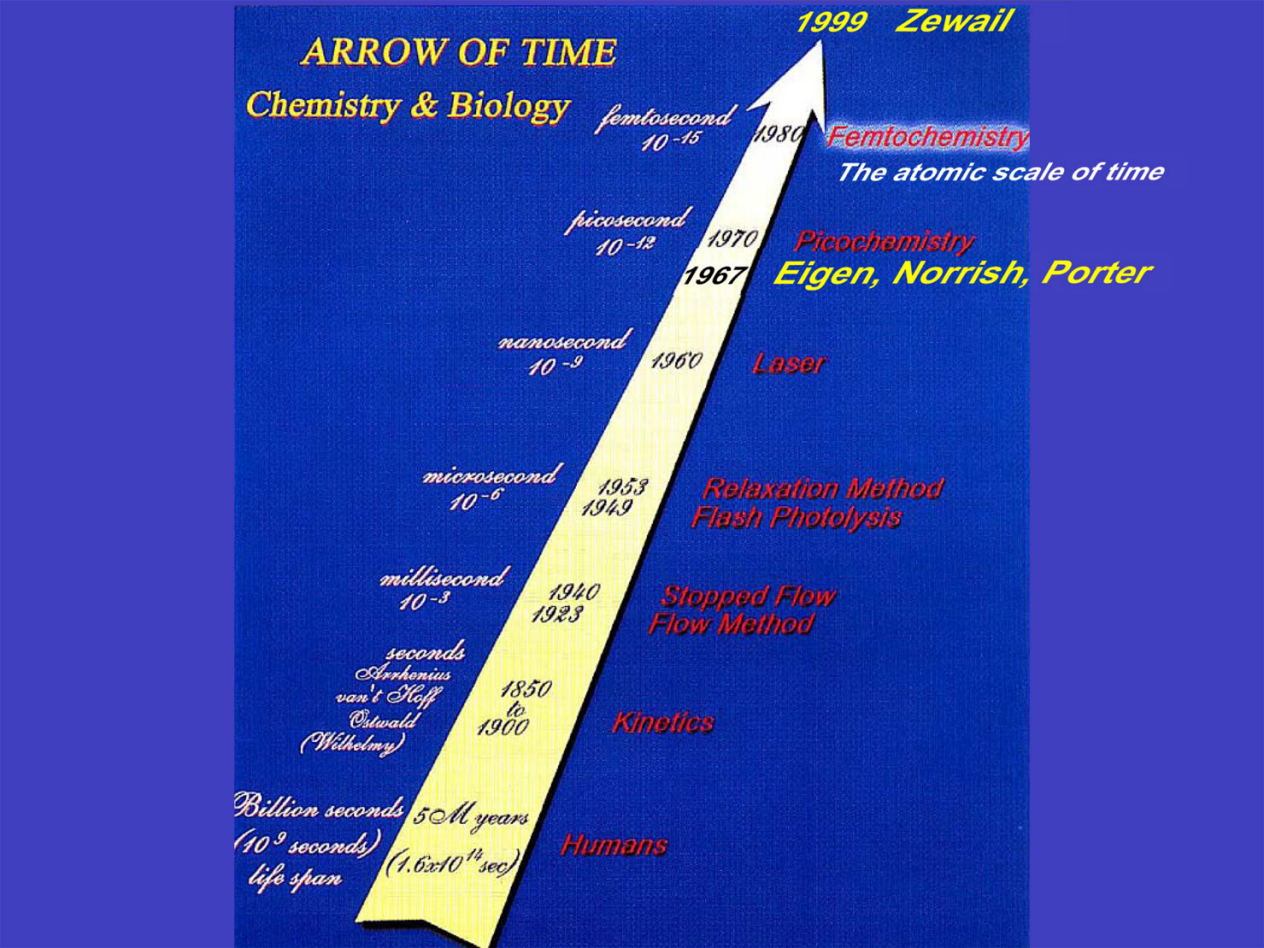
The arrow of time in Chemistry and Biology, as regularly shown by Ahmed Zewail in his talks during the 1990s. Reproduced with permission from A. H. Zewail, Angew. Chem. Int. Ed. **39**, 2587–2631 (2000). Copyright 2000 John Wiley and Sons.

Prior to the Nobel Prize, in many talks, Ahmed Zewail used to show his famous slide with the arrow of time (Fig. 1), which we have slightly modified here to add him. In 1889, Arrhenius presented a description of how chemical reactions vary as a function of temperature. His well-known formula describes the reaction rate K(T) in s^−1^ as a function of temperature

$$K\left(T\right)=A\;\;\text{exp}\;\left(-E_{a}/\text{kT}\right),$$

where k is the Boltzmann constant, T is the temperature (in Kelvin) and E_a_ is the so-called activation energy, i.e., the height of the barrier up to a hypothetical state called by Arrhenius, the “activated complex.” The Arrhenius equation has been used with success by chemists and physicists to describe kinetic processes in a large class of media. It reflects the evolution of populations of species from reactants to products. Experimentally, the 1920s saw the implementation of flow and stopped flow methods allowing to follow chemical reactions on time scales of milliseconds.

The early 1930s were a period of intense activity aimed at understanding and describing atomic collisions and chemical reactions. In a short period of time (1932 and 1933!), Landau (in the Soviet Union), Zener (in England) and von Stueckelberg (in the United States),^27–30^ developed the celebrated Landau-Zener-Stueckelberg formalism, which is very commonly used to describe the hoping probability in non-adiabatic transitions at an avoided crossing between two potential curves. Around the same time, Eyring and Polanyi ^31,32^ developed the first potential energy surface for the H_2_ + H → H + H_2_ reaction, and in 1936, Hirschfelder *et al.*,^33^ performed the first trajectory calculation with femtosecond steps using the then computing capabilities. These developments would mark the birth of “reaction dynamics” that introduced the concept of potential energy surfaces with dynamics occurring on them: the path of a reaction from reactants to products, through valleys and mountains, with the transition state at the saddle point. In those years, Eyring,^34,35^ Evans and Polanyi^36,37^ formulated the transition-state theory. Using statistical mechanics, they derived an analytical expression for the pre-exponential factor A in the Arrhenius equation, with the rate constant being proportional to a “frequency” factor for the passage through the transition state. This factor is ∼10^13^ Herz, the order of magnitude of molecular vibrational frequencies!^38^

Another concept that would later be exploited by Ahmed Zewail in his experiments and would be crucial to probing the structural dynamics of molecular systems stems from the 1920s, when the foundations of quantum mechanics were being laid down. In 1926, Erwin Schrödinger introduced the concept of “wave groups” in order to connect the quantum and classical descriptions.^39^ In 1927, Ehrenfest published the theorem that bears his name,^40^ and outlines the regime for the transition from a quantum to a classical description: the quantum expectation values behave classically in the limit of large quantum numbers. The use of “wave groups” in Physics (and, obviously in Chemistry) remained limited to a few Gedanken experiments, as it was not possible to experimentally generate wave groups (now called wave packets). The field had to wait several decades in order to reach the required time resolution.

**FIG. 2.**
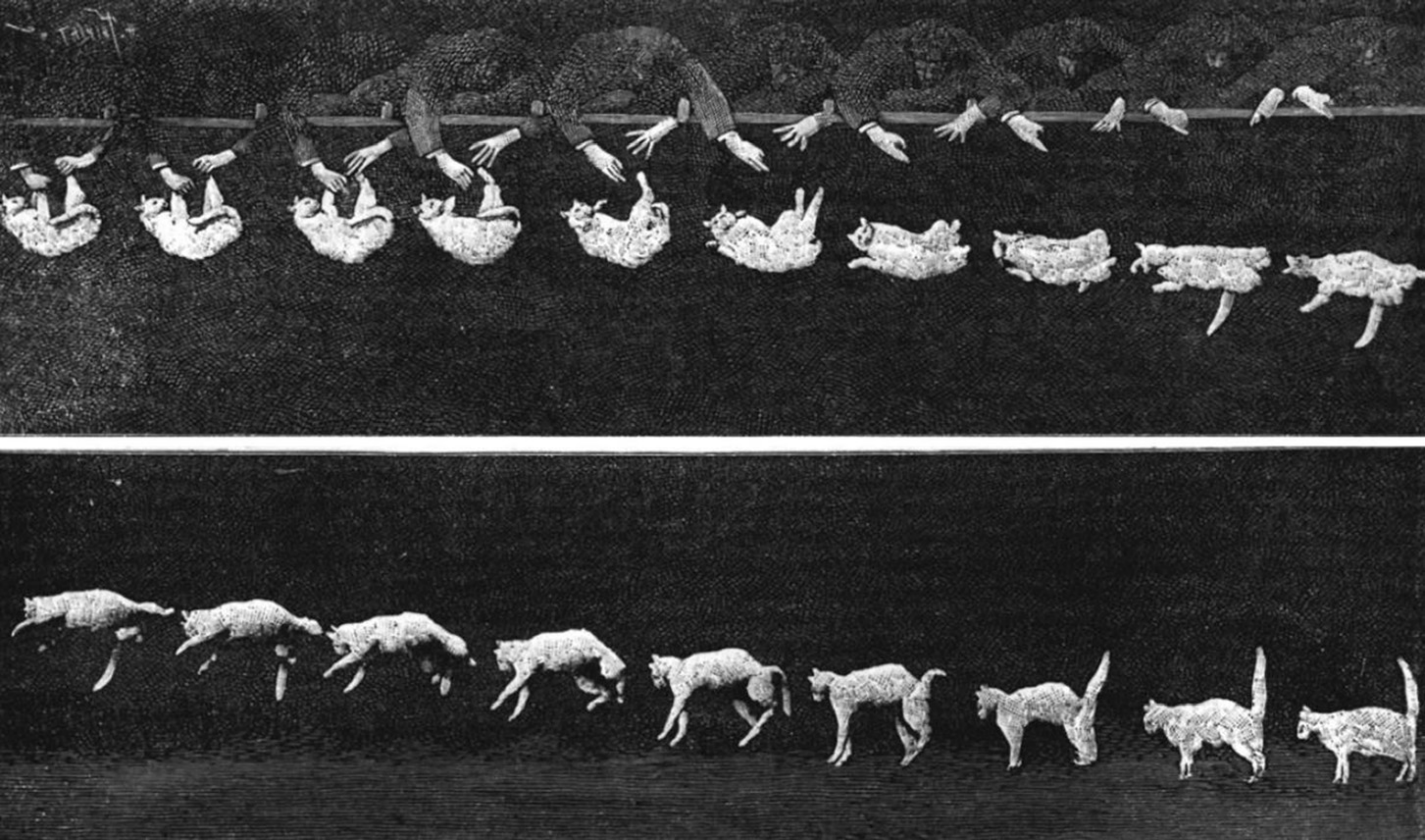
This type of snapshot of a falling cat was for the first time captured by chronophotography in 1894 by the French physiologist Etienne-Jules Marrey who developed the shutter camera. Reproduced with permission from E.-J. Marrey, La Nature **1894**, 1119. Copyright 1894.^41^

The time scales of chemical and physical transformations in matter span several decades, and the required time resolution depends on the scientific question one needs to address. Animal motion requires typically a millisecond resolution, and Ahmed Zewail used to show Muybridge's famous snapshots of the galloping horse to illustrate the connection to molecular motion. However, the first snapshots of animal motion to be taken by a single photographic apparatus were made by the French anatomist and physiologist, Etienne-Jules Marrey,^42^ who developed the shutter camera. He was interested in animal motion, and one of the famous cases he filmed was that of the cat being released paws up and lands on them at the end of its fall after performing a double-isomerization (Fig. 2). Scaling the size of the cat down to that of a chemical bond and scaling the time resolution down by the same factor bring us in the sub-picosecond regime. Thus, as the length scale of dynamical processes decreases, e.g., protein motions, acoustic waves, molecular rotation, molecular vibrations, there is need for higher and higher temporal resolution spanning the range from nanoseconds (ns) to femtoseconds (fs).

The long standing efforts to improve the time-resolution in dynamical studies of matter were the works of many researchers (Fig. 1). Porter and Norrish introduced the Flash Photolysis technique at the end of the 1940s, which allowed the millisecond time resolution.^43^ With his legendary taste for historical precision, Ahmed Zewail recalled in his Nobel Lecture^44^ that the first pump-probe experiment in the tens of nanosecond time domain(!) had been carried out in 1899 by the French physicists Abraham and Lemoine,^45^ who demonstrated the Kerr response of liquid CS_2_. In the early 1950s, by exposing a chemical solution to heat, pressure or an electrical shock, Eigen achieved the microsecond temporal resolution.^46^ Eigen, Porter and Norrish were credited with the Nobel Prize for Chemistry in 1967 for their discoveries, but the real game changer in terms of time resolution was the discovery of the laser in the early 1960s, thanks to the works of Javan,^47,48^ Basov, Prokhorov,^49,50^ and Townes and Schawlow.^51^ Soon after the birth of the laser, pulsed nanosecond, then picosecond lasers appeared.^52,53^ However, even on the short picosecond time scale, molecular states reside in eigenstates (the static limit), only one evolution is observable: the change of population with time. Hence, one is still dealing with *kinetics*. To enter the realm of *dynamics*, i.e., the time scale in which atoms evolve as rigid spheres, the atomic scale of time (as Ahmed Zewail named it), i.e., the femtosecond was needed. Laser technology had to wait the mid-1980s to see the advent of femtosecond laser technology.^54–57^ It became possible to probe molecular motion in real-time. Indeed, the vibrational periods of molecules span a large range: from ∼8 fs for diatomic hydrogen to ∼300 fs for diatomic iodine.

## II. THE FIRST REVOLUTION: FEMTOCHEMISTRY

**FIG. 3.**
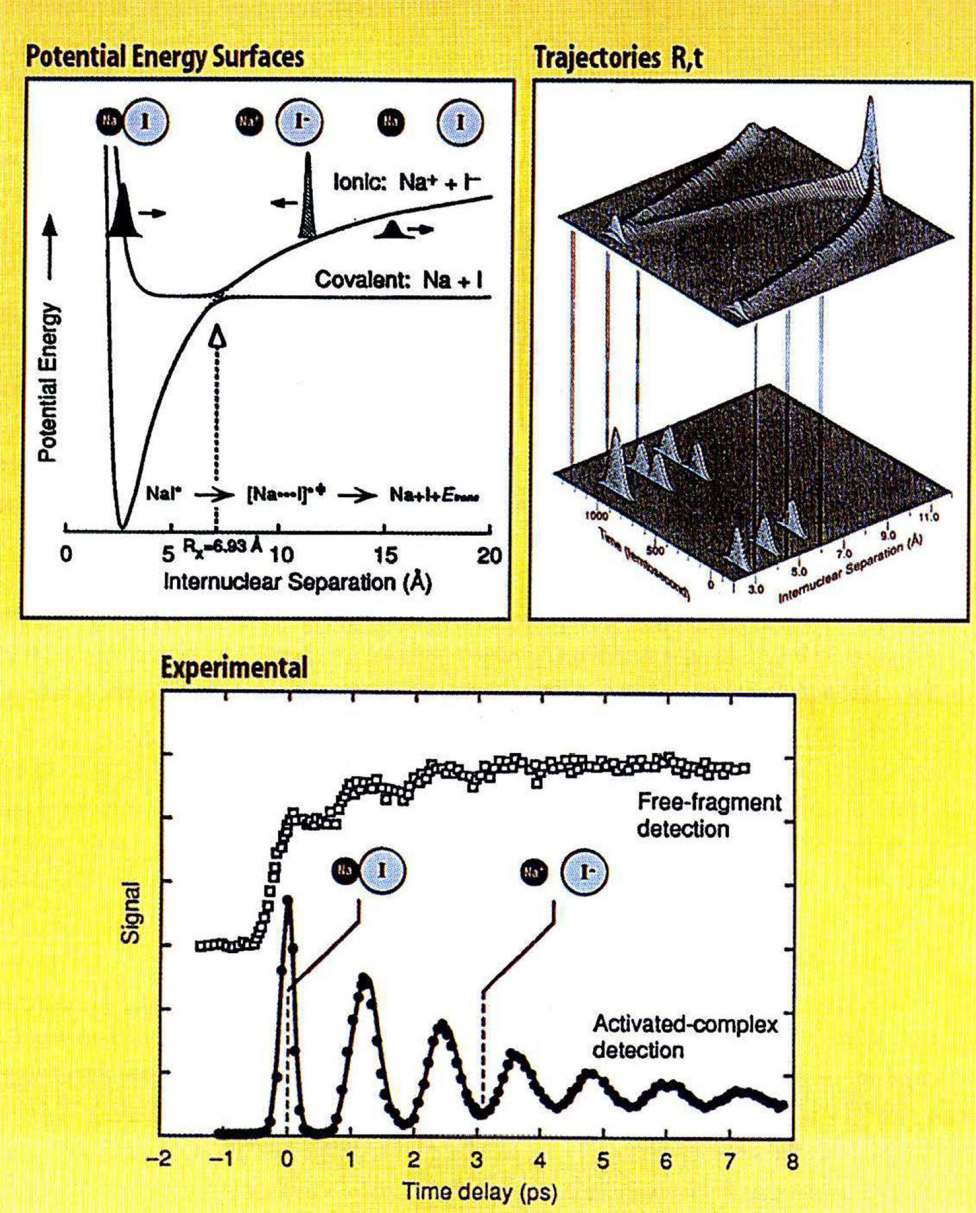
Femtochemistry of NaI. The vibrational wave packet created on the excited state evolves between the covalent and ionic parts of the potential curve. Upon passage by the point of crossing of the potential curves, the decay of the transient [Na…I] species and the build-up of Na (bottom panel) were observed. Reproduced with permission from A. H. Zewail, Angew. Chem. Int. Ed. **39**, 2587–2631 (2000). Copyright 2000 John Wiley and Sons.

With ps pulses, a number of researchers managed in the mid-1980s to observe Rydberg (electronic) wave packets since the energy spacings in high-n Rydberg states can be tiny enough that their inverse time is several ps. These electronic wave packets were observed mostly in alkali atoms,^58–62^ and also in solids, where the hydrogen-like excitonic series was exploited.^63^ To create vibrational wavepackets in molecular systems, shorter, femtosecond duration, pulses were needed. This temporal resolution is sufficiently short to “freeze” the nuclei at a given internuclear separation, i.e., the time resolution is much shorter than the vibrational (and rotational) motions such that the wavepacket is prepared, highly localized, with the structure frozen on the excited state potential surface (Fig. 3). This ultrashort perturbation does not violate the uncertainty principle. On the contrary, a wavepacket is a coherent superposition of eigenstates and therefore, the system is coherently prepared. Because of this coherent preparation, the high temporal resolution implies a spatial resolution, so that one is able to monitor the evolution of the system at the atomic resolution!

With this powerful tool in hand, Zewail and co-workers could observe the dynamical processes of bond-breaking, bond making and of the cornerstone of reactivity, the transition state, with atomic scale resolution. To take a picture of a simple chemical reaction, Zewail and co-workers used the well established method of flash photolysis^64^ in which a first femtosecond pulse (called the pump pulse) excites the system, triggering a dynamical process therein (dissociation, vibrations, isomerization, electron transfer, etc.), while a second, so-called probe pulse, monitors the evolution of the system at a variable time delay with respect to the pump pulse, either by absorption between excited states of the system, or a fluorescence or ionization signal that this absorption may give rise to.

For the first time, it became possible to probe in real-time the motion of atoms inside molecules, via their wavepacket dynamics, and monitor the chemical reactions and the transition state. Key to the impact of Zewail's seminal contribution in Science is his systematic strategy of studying in a stepwise fashion systems of ever greater complexity, starting from the simplest ones, thus demonstrating in a very didactic and convincing way the power of the new methodology and the insight it delivers into the dynamics of molecular phenomena. This is a crucial point about Zewail's contribution to Science, because while others used femtosecond laser simultaneous to him around 1987–1988,^65,66^ and even observed vibrational wave packet dynamics,^65^ the complexity of the system being investigated was too high to convey a clear message that would convince the community.

Under Zewail's leading role, the first age of femtochemistry was marked by the study of simple molecular systems, in order to establish the experimental methods and the theoretical concepts.^67,68^ The experiment that marked the birth of Femtochemistry by Zewail and co-workers was published in 1987–1988.^69,70^ It dealt with the dissociation of cyanogen iodide (ICN), in which the appearance of a free CN fragment was found to occur in about 200 fs. The observation of vibrational wave packets and their use to probe non-adiabatic transitions between electronic states of molecules were strikingly well exemplified in the famous case of the NaI molecule (Fig. 3).^71^ He then demonstrated how one can retrieve the details of the anharmonic parts of the excited state potential with a precision better than that of conventional spectroscopy.^72,73^ Thus, what is a very challenging task in frequency domain experiments, due to the high density of molecular vibrational-rotational states, becomes an easy one in time-domain experiments.

He then addressed an issue that had occupied chemists for many years: why are certain chemical bonds more reactive than others? What happens if a molecule has two equivalent bonds? Will they break simultaneously or sequentially? He tackled this problem by studying the photdissociation of diiodotetrafluoroethane (C_2_F_4_I_2_) into tetrafluoroethane and two iodine atoms, and found^74^ that the two C-I bonds break sequentially, rather than in a concerted fashion. This experiment would later play a crucial role in the implementation of ultrafast electron diffraction (UED).

Studying bimolecular reactions in real-time was a challenge because of the issue of clocking the encounter between reactants. Zewail implemented a judicious femtosecond experiment to examine analogues of the activated complex involved in bimolecular reactions,^75^ by using molecular beams. He thus generated a weakly H-bonded IH···CO_2_ complex in the ground state and by dissociating the H atom from HI, the reaction with CO_2_ leads to an HOCO intermediate whose evolution till its decay into HO and CO products could be probed in real-time. This reaction is of crucial importance in combustion chemistry.

Increasing the complexity of the reactions he investigated, he attacked the classic case of ring opening of cyclobutane to yield ethylene or the reverse process, typical of the Woodward-Hoffman reaction. The core issue here is the competition between two mechanisms: a concerted one-step process versus a two-step process with an intermediate. An Arrhenius type treatment of the reaction is unable to distinguish between the two schemes, and one actually sees only an activation barrier. By combining femtosecond spectroscopy with time-of-flight mass spectrometry and molecular beams, Zewail and co-workers clearly established the existence of a diradical as a distinct molecular species on a subpicosecond time scale.^76^ Nearly all types of reactions in organic chemistry (Diels-Adler,^77,78^ Norrish type,^79^ Bodenstein type^80,81^ and many others^82–88^) were scrutinized by Zewail and his group. This allowed him to define the mechanistic concepts crucial to the understanding of the nature of chemical bond changes. In a way, the whole debate of concerted vs non-concerted that had dominated organic photochemistry boils down to a question of time scale as before the advent of femtosecond spectroscopy and Zewail's contributions, and it was not possible to distinguish the reaction pathways.

The next challenge that he took up was to investigate chemical dynamics in condensed phases. The competition between solvent fluctuation times and timescales of reactive motions is crucial if one wants to avoid randomization of energy along a specific reactive coordinate. The interplay between these time scales (coherent versus diffusive) was first addressed by him using femtosecond spectroscopy. He thus investigated various reactions ranging from barrier crossing events,^86^ over rotation of molecules^88^ to bond breaking events.^89^ Contrary to the expectation of ultrafast energy randomization, coherent nuclear motion can be maintained in molecular systems in solution. A fact that was repeatedly confirmed for molecules in solutions or in crystals^89–95^ and for biological chromophores in proteins.^96,97^

Elementary chemical reactions like cis-trans isomerizations and ligand dissociation are the basis of the function of many biological photoreceptors (retinal in vision and in bacteriorhodopsin; linear tetrapyrroles in phytochrome; p-coumaric acid in the photoactive yellow protein, etc.) and enzymes (myoglobin, hemoglobin, cytochrome, etc.). The ability to visualize vibrational motion in a protein enables studies of the relation between nuclear motion and biological function. As an example, it is known that hydrogen bonds bind the double-stranded DNA helix and determine the complementary of the pairing. By carrying out femtosecond pump-probe studies on model base pairs (e.g., 7-azaindole), Zewail's group^98,99^ tracked the dynamics of hydrogen bonds and the ensuing tautomerization, identifying different time scales of structural relaxation in the initial pair, vibrational relaxation and cooling of the tautomer. These studies have yielded a detailed molecular picture of the nuclear dynamics, and are model studies of the fundamentals of chemical reactivity in biological functions.

Finally, Zewail was one of the first to investigate the dynamics of water around proteins using biological fluorophores (in particular, tryptophan) as probes of the medium.^100–102^ By time resolving the ultrafast fluorescence decay of the fluorophore at the surface of the protein and comparing it to that in the bulk, he concluded that the water shell around the proteins consists of barely two monolayers of molecules. These studies sparked off an intense activity aimed at elucidating the role of the hydration shell around proteins.^103^

This far from exhaustive review of the first phase of Ahmed Zewail's carreer leading to the Nobel Prize in 1999 shows how he opened the field and popularized ultrafast science to a broad community of scientists. If the first audience he targeted was the chemical physics community, there is no doubt that his second audience included all those interested in the phenomenology associated with the condensed phases, materials science and biology.

## III. THE SECOND REVOLUTION: STRUCTURAL DYNAMICS WITH ELECTRONS

In optical pump-probe experiments, both the excitation and the probing rely on an *a priori* knowledge of the spectroscopy (energy, intensity and lineshape of bands) of the system under study. However, optical-domain spectroscopic observables can be translated into atomic coordinates, only in very few cases, such as small diatomic or triatomic molecules, for which potential curves or potential surfaces are available. Therefore, already in the early days of Femtochemistry, it was clear to Zewail and many in the ultrafast science community that optical-domain probe pulses needed to be replaced by pulses of short wavelength radiation, i.e., electrons or X-rays, in order to move the famous tools of structural determination (X-ray or electron diffraction and X-ray absorption spectroscopy) into the time domain.

While most of the community adopted the approach based on the use of ultrashort pulses of X-rays,^104–107^ Ahmed Zewail was the only scientist to adopt the approach with ultrashort electron pulses. This choice was extremely judicious, and turned out to be far reaching, for the following reasons: (i) as already mentioned, the scattering cross-section of electrons is 5–6 orders of magnitude larger than that of X-rays. Thus, electrons can be used to probe low density media (e.g., gas phase molecules, adsorbates on surfaces) and light elements (very important for chemical and biological systems); (ii) the energy deposited per scattering event is 3 orders of magnitude smaller than in the case of X-rays; (iii) electron optics make beams of electrons easier to manipulate than X-rays, and the tools of electron microscopy were readily available.^108–110^

Within a few years from his seminal work^69,70^ that marked the birth of Femtochemistry, he published a series of papers that laid down the fundamental concepts and methodological principles of ultrafast electron diffraction (UED),^111–115^ and achieved the first scientific results, with a resolution of a few to a few tens of picoseconds, on small molecules,^115^ metal-carbonyls^116–118^ and on the determination of transient structures of chemical reactions.^117,119–121^ A striking demonstration was his study of photodissociation dynamics of C_2_F_4_I_2_, which showed how UED could address questions (the structure of a short lived intermediate) hitherto unanswered by other techniques, and warned about the use of quantum chemical (equilibrium) structure calculations to interpret dynamics. This type of study has only recently been achieved using ultrashort X-ray pulses from a free electron laser.^122^

When one of us was invited to compose News&Views articles based on the Williamson-Zewail article,^111^ he was led to conclude^123^ that in view of the major advance in time resolution of electron-probe methods, a new era in ultrafast electron-crystallography was about to dawn. The subsequent brilliant work by Zewail, some of it described here, and also in references,^124–127^ has triumphantly vindicated the above prediction.

Zewail then moved on to determining the structures of complex molecules far from their equilibrium configuration.^128,129^ A corollary of this study was the determination of transient structures in radiationless transitions.^121,130–133^ He thus showed that the “channel three” process in polyatomic molecules, which was assumed to be a non-radiative relaxation to high vibrational levels of the ground state, is characterized by structural modifications of the molecule and is thus photochemical in its nature. It is useful to stress that all the above studies were carried out on molecules consisting of light elements, and there is no way the results harnessed by UED could have been obtained by other techniques, least of all X-ray ones. A striking example of this unique ability of UED was demonstrated by Prof. Zewail in his study of hydrogen-bonded structures in resonant and tautomeric reactions,^134^ on the elimination reaction of acetylacetone,^135^ and on the release of NO from nitrobenzene.^136^

Around 2003–2004, he achieved another great leap forward: that of investigating condensed matter by UED and ultrafast electron crystallography (UEC).^137^ In a breakthrough experiment, he determined the structural change of interfacial water on a hydrophilic surface, and its timescale, upon impulsive heating with a short laser pulse.^138^ He also determined the structural changes of laser-excited surfaces,^139^ as well as of laser-excited adsorbates on surfaces,^137^ and the importance to biology, of phospholipids^140^ and fatty acid bilayers.^141,142^

In his work on the long-enigmatic insulator-metal phase transition of VO_2_,^143–145^ he was able to probe the lattice structure changes with an unsurpassed degree of detail, showing that there is a stepwise mechanism for atomic motions. The application of these techniques to phase transitions in superconducting materials,^146^ in nanostructures and heterostructures,^145,147–149^ and in graphite,^150^ has delivered a completely new view of the underlying mechanisms, in particular the chemical bond changes and their time scales.

**FIG. 4.**
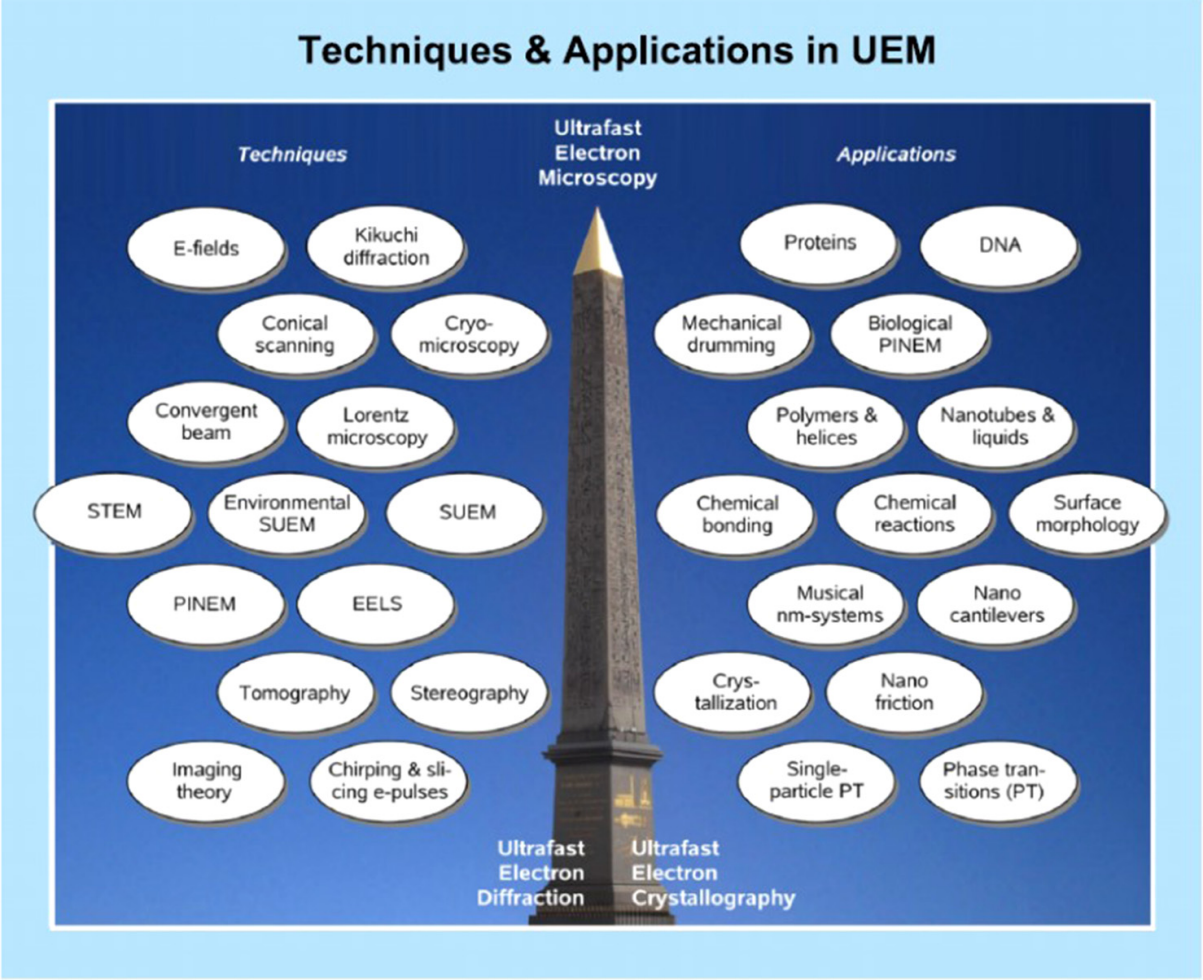
Developments and the range of applications of 4D UEM. On the left, the techniques developed over the last decade are indicated. On the right, the various applications made using these techniques are presented. At the bottom of the Egyptian obelisk the two “precursors,” UED and UEC, are shown, and the “ultimate” development of UEM is displayed at the apex of the obelisk. From A. H. Zewail, *4D Visualization of Matter: Recent Collected Works*. Copyright 2014 Imperial College Press. Reproduced with permission from Imperial College Press.^159^

The next great turn in Ahmed Zewail's implementation of new electron-based tools is his development of *real-time* and *real-space* ultrafast electron microscopy (UEM),^143–145,151–153^ which represents a real revolution in structural dynamics.^125^ Zewail devoted the last 10 years or so of his life to applying and further developing this method.^154^ A striking example of ultrafast *real-time* and *real-space* imaging using UEM and UEC is his work on gold and graphite.^155^ He went on developing new tools, which each by itself could, and in some case did, evolve into a separate new field.^154^ One of the last studies he conducted prior to his passing was carried out in conjunction with one of us on the study of 4DEM in the study of single-site Ti photocatalysists,^156^ measuring the trapping time of the electron at pentacoordinated Ti centres.^157^ This work was described by Harry Gray as a ground-breaking contribution to photocatalysis.^158^ Figure 4 is adapted from a recent compilation of Ahmed Zewail's work, and it shows the diversity and versatility of time resolved electron-based methods he pioneered.^159^ Among the methods shown in this figure, we would like to stress just a few.

**FIG. 5.**
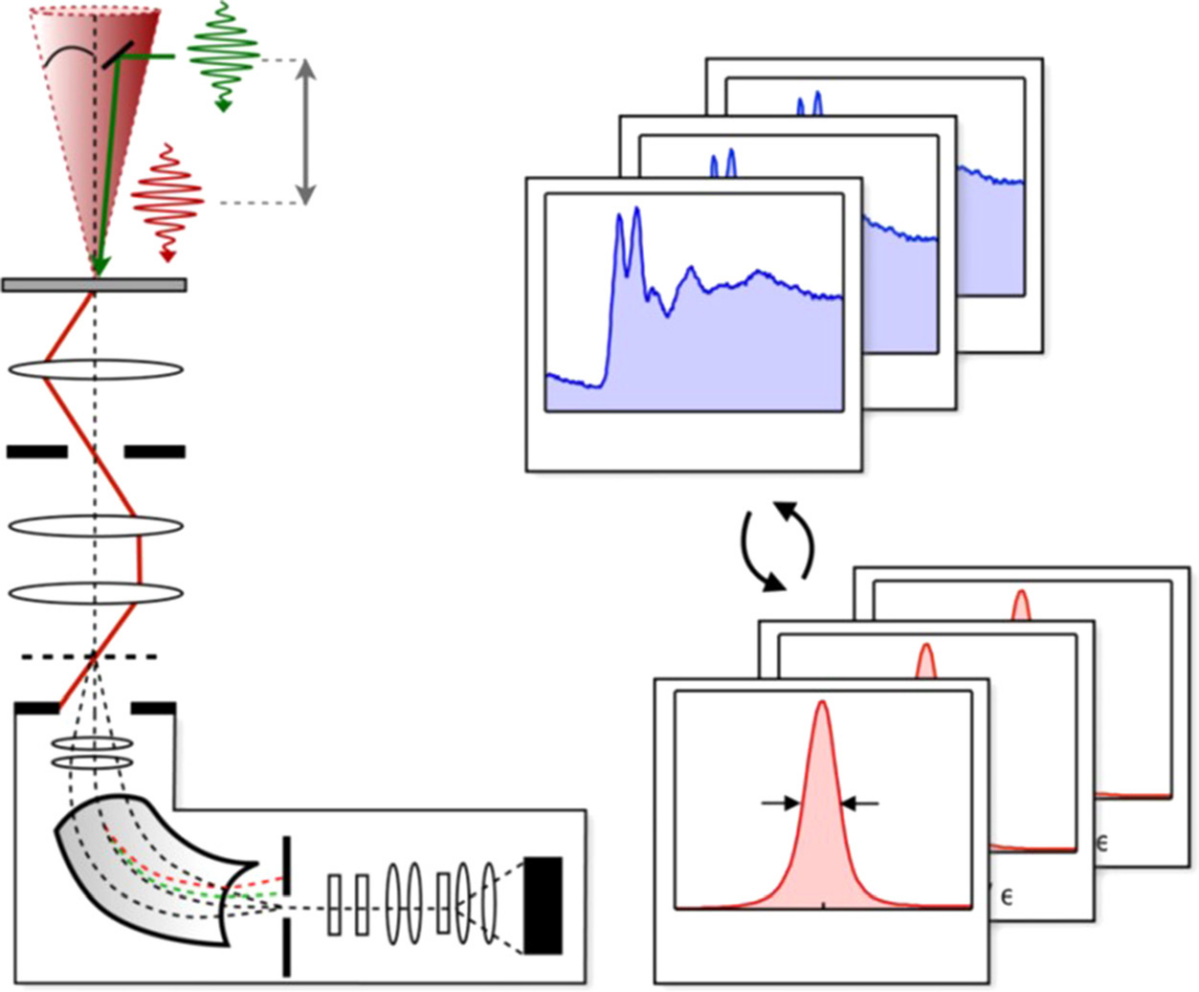
Ultrafast core-loss spectroscopy. The short laser pulse (hν, green) initiates the structural change at t = 0 and acts as a clocking pulse. The short, converged electron packet with convergence semi-angle a (red) illuminates a small area (∼100 nm) of the graphite nanometer-thin film and is time delayed with respect to the clocking pulse (Δt). Electron propagation paths are schematically denoted as red lines. Electron energy-loss spectra are recorded by collecting the diffraction pattern of the converged electron beam at the spectrometer entrance aperture and dispersing the pulsed electrons using a post-column spectrometer. The energy resolution (∼2 eV) is determined from the full-width-at-half maximum (FWHM) of the ZLP, corresponding to elastically scattered electrons passing through the aperture. Energy drifts are carefully corrected by alternatingly recording core-loss and zero-loss spectra and using the position of the ZLP to adjust the energy axis. Reproduced with permission from van der Veen, Penfold, and Zewail, Struct. Dyn. **2**, 024302 (2015). Copyright 2015 AIPP.^162^

He pioneered time-resolved Electron Energy Loss Spectroscopy (EELS), which he successfully applied to the study of graphite (Fig. 5).^125,160,161^ More recently, he extended ultrafast EELS to the study of deep core levels.^162^ This brought “chemical selectivity” into the realm of electron-based techniques, and combining ultrafast EELS with ultrafast UEC^148^ delivers the full information in Fourier and energy space.^161^

**FIG. 6.**
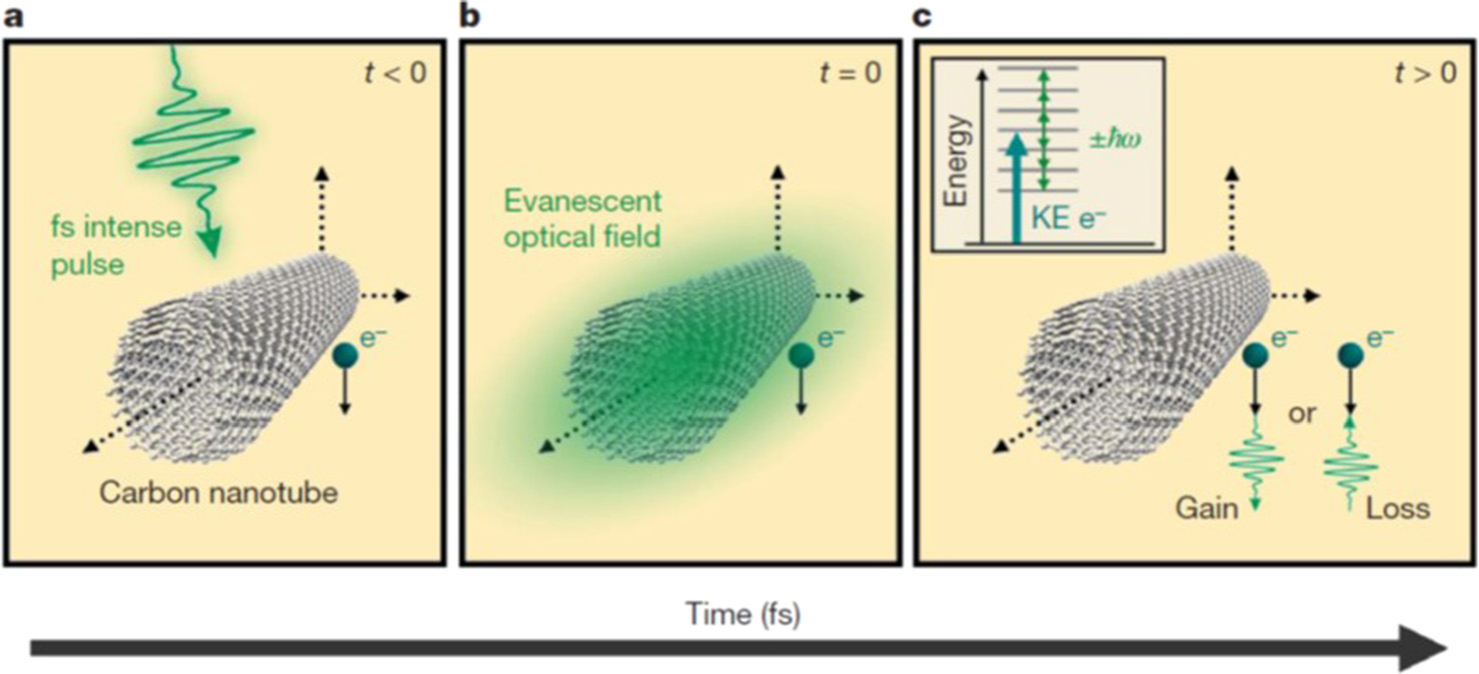
Physical depiction of the interaction between the electron, the photon and the evanescent field at the origin of photo-induced near field electron microscopy (PINEM). (a) When the electron packet arrives at the nanotube before the femtosecond laser pulse (t, 0); no spatiotemporal overlap occurs. (b) The electron packet, the femtosecond laser pulse and the evanescent field are at a maximum overlap at the carbon nanotube. (c) Illustration of the process during and immediately after the interaction (t > 0) when the electron gains/loses energy equal to integer multiples of femtosecond laser photons. The inset shows the possible final energies in the continuum due to the free–free transitions between the imaging electron and the photons in the femtosecond laser pulse (KE = kinetic energy). Reproduced with permission from Barwick *et al.*, Nature **462**, 7275 (2009). Copyright 2009 Nature Publishing Group.^155^

Another important discovery of Ahmed Zewail is Photoinduced Near Field Electron Microscopy (PINEM), which has developed into a major tool in nanoscience. In PINEM, an ultrashort optical pulse of wavelength λ excites a nanostructure of a characteristic dimension d  ≪  λ, giving rise to the evanescent near-field that turns into electromagnetic waves in the far-field. Simultaneously, an ultrashort electron pulse images the field through inelastic scattering of ultrafast electrons (Fig. 6). It is impossible for the electron and the photon to interact in the absence of the nanostructure, which provides spatial localization sufficient for momentum conservation. The extent of spatial localization in the longitudinal direction needed for momentum conservation can be estimated by considering the uncertainty relationship between Δz and Δp. PINEM enables visualization of the spatiotemporal dielectric response of nanostructures, of plasmonic fields and their spatial interferences, imaging of low-atomic-number, nanoscale materials, characterization of ultrashort electron packets, and imaging of various biological assemblies.^163–168^

This brief overview of Zewail's seminal contribution to the field of structural dynamics would not be complete without mentioning his constant development of new concepts and methodologies, which still promise breakthroughs in the future. For example, his early idea of generating attosecond pulses of electrons,^144,150,169–171^ which is promising for the study of electron dynamics of the chemical bond, is so far limited by the fact that only electromagnetic attosecond pulses in the XUV domain are used that have huge energy bandwidths (typ. 30 eV), impeding any selectivity and making the study of valence electrons difficult.^172^

In summary, it is indisputable that in a time span of less than 15 years, Ahmed Zewail's achievements in UED, UEC and UEM had led to a completely new way of looking at a large class of phenomena in a wide variety of systems. Whether we talk about reactions in complex gas phase molecules, phase transitions in thin films and biological samples, phase transitions in solid materials, nanostructures or heterostructures, etc., thanks to Ahmed Zewail, there is a striking difference in our understanding of these phenomena between before and after his breakthrough experiments.

## IV. CONCLUSIONS

Through his pioneering works in the field of Femtochemistry, Ahmed Zewail has opened up new avenues in chemistry, biology and materials science that could not have been foreseen 30 years ago. In March 1993, at the Berlin Conference on Femtochemistry, the 1967 Nobel Laureate Sir George Porter observed: “The study of chemical events that occur in the femtosecond time scale is the ultimate achievement in half a century of development and, although many future events will be run over the same course, chemists are near the end of the race against time”. If this is true as far as nuclear dynamics is concerned, sub-femtosecond pulses or attosecond pulses (1 attosecond = 10^−18 ^s) are now proving useful to probe the motion of the electron and of valency that play such a crucial role in chemistry.^173,174^

The translation of traditional electron-based structural tools into the ultrafast time domain was also one of Ahmed Zewail's major achievements. Many believed that he was well on the way to a second Nobel Prize. His legacy is now being picked up by many young and brilliant scientists, several of which have been former members of his group, as well as by many other scientists.^175–177^ The awareness brought about by the spectacular achievements in ultrafast electron-based science have surely cross-fertilized the field of ultrafast X-ray science, which has been marked by no less spectacular achievements.^178^ This is becoming more evident with the development of X-ray free electron lasers (XFELs),^179–181^ which are based on high energy electron accelerators. We are even witnessing a convergence of the two branches of ultrafast structural dynamics with several groups increasingly using the MeV electron pulses from XFELs, which have the advantage of overcoming space charge due to their relativistic speeds.^182,183^ Schemes are also being proposed to combine the MeV electrons with the X-ray pulses from XFELs for the study of matter, with element- and orbital-selective excitation.^184^

We conclude this tribute with the words of Professor Harry Gray, who as Chair of the Chemistry Faculty at Caltech appointed Ahmed Zewail as a young assistant professor: *Rest in Peace Ahmed. You will be remembered as long as Science is done on Planet Earth*.^158^
